# Reply: Critical commentary on the study comparing TIPS, tunneled peritoneal catheter, and ascites pump in refractory ascites

**DOI:** 10.1097/HC9.0000000000000726

**Published:** 2025-05-23

**Authors:** Sarah L. Schütte, Benjamin Maasoumy, Tammo L. Tergast

**Affiliations:** 1 Department of Gastroenterology, Hepatology, Infectious Diseases and Endocrinology, Hannover Medical School, Hannover, Germany; 2 German Center for Infection Research (DZIF), Hannover–Braunschweig, Germany

We highly appreciate the interest of Dr Hassan in our study.^1^ Refractory ascites (RA) poses a clinical challenge in the care of patients with decompensated advanced chronic liver disease, especially in times of organ shortage. We agree that in a perfect scenario, a randomized controlled trial would deliver the best scientific evidence to ultimately compare the ascites pump (AP), peritoneal catheter (PeCa), and TIPS. However, the obstacles to conducting such a study are significant. TIPS insertion targets the underlying pathomechanism, and it is therefore to be expected that the TIPS procedure is superior to AP and PeCa, which both are more symptomatic treatment approaches.^1^,^2^,^3^,^4^ It may therefore be questionable to withhold TIPS placement from patients who would be eligible, since it is recommended as a first-line treatment in patients with RA. In fact, only patients with contraindication for TIPS or ascites persistence after TIPS underwent AP or PeCa implantation in our study. Some bias is therefore to be expected, even after propensity score matching. Nonetheless, there is only very limited data that directly compares clinical outcomes between all devices. Hence, reporting of these data is of high interest, even if it is to strengthen TIPS as a first-line treatment option in patients with RA.

Furthermore, there was currently no data available comparing PeCa and AP. Especially, AP implantation was limited to very few centers in Europe. As of now, AP implantation is no longer available in Europe, and PeCa implantation is a rather new procedure to treat RA. Still, data comparing both devices are of interest since both offer potential palliative treatment options, which are highly warranted in decompensated advanced chronic liver disease without the option for liver transplantation or TIPS.^2^,^3^,^4^ However, randomized trials comparing paracentesis with albumin and PeCa are currently being conducted, also raising important quality of live data.^4^
